# Pilot study on seasonal variability in microbial contamination in the developing tourist region of Mindo, Ecuador: a comparative analysis of the Saguambi, Mindo, and Canchupí Rivers

**DOI:** 10.1186/s13104-025-07205-3

**Published:** 2025-04-10

**Authors:** Milena Naomi Martínez-Játiva, Pamela Borja-Serrano, Hugo Valdebenito, António Machado

**Affiliations:** 1 Universidad San Francisco de Quito USFQ, Colegio de Ciencias Biológicas y Ambientales COCIBA, Instituto de Microbiología, Laboratorio de Bacteriología, Quito, 170901 Ecuador; 2 Universidad San Francisco de Quito USFQ, Colegio de Ciencias Biológicas y Ambientales COCIBA, Herbarium of Economic Botany of Ecuador, Quito, 170901 Ecuador; 3 Universidade dos Açores, Faculdade de Ciências e Tecnologia, Departamento de Biologia, Centro de Biotecnologia dos Açores (CBA), 9500-321 Ponta Delgada, Portugal

**Keywords:** Microbial contamination, Water quality, Public Health risk, Pathogens, One Health approach, Sustainable tourism

## Abstract

**Objective:**

This study aims to evaluate the microbial load in the Saguambi, Mindo, and Canchupí Rivers in Mindo (Ecuador) by quantifying bacteriological indicators (*Escherichia coli* and total coliforms) and identifying pathogenic microorganisms (e.g., *Giardia*, *Cryptosporidium* spp., *Helicobacter pylori*, *Mycobacterium tuberculosis*, and *Mycobacterium leprae*) using molecular techniques. This assessment aims to establish the potential risk associated with the consumption and recreational use of these water sources.

**Results:**

A total of 36 surface water samples were analyzed in this study, with 12 samples collected per river (Saguambi, Mindo, and Canchupí). Sampling was conducted in duplicate at two collection points per river (before and after the community) across three seasons (dry, rainy, and transitional), resulting in 4 samples per river per season. All samples showed consistently high microbial levels exceeding international guidelines at most collection points across the three rivers. The Canchupí River exhibited the highest *E. coli* and total coliform counts during the dry season, with values of 1.50 × 10^7^ and 1.79 × 10^7^ CFU/100 mL, respectively. The Saguambi River showed the highest *E. coli* levels in the transitional season (9.42 × 10^4^ CFU/100 mL). The Mindo River peaked in *E. coli* (7.15 × 10^5^ CFU/100 mL) and total coliforms (5.85 × 10^5^ CFU/100 mL) after the community. Molecular analysis identified *M. tuberculosis* in all rivers year-round. *M. leprae* was found in the Saguambi and Mindo Rivers, and *H. pylori* was identified in both Mindo and Canchupí Rivers. *Giardia* and *Cryptosporidium* parasites’ detection varied among rivers and seasons.

**Supplementary Information:**

The online version contains supplementary material available at 10.1186/s13104-025-07205-3.

## Introduction

Water pollution from untreated wastewater poses significant environmental and public health issues globally, with 80% of wastewater discharged untreated into water bodies. In Ecuador, the reliance on surface water as drinking water exacerbates this problem [1]. In rural areas like Mindo, wastewater is directly discharged into the Canchupí River [2], and there is inadequate drinking water coverage [3]. Mindo, located 70 km from Quito in the Mindo-Nambillo protected forest [4], relies heavily on tourism, particularly river-based activities, as its main economic source [5]. It has a diverse river network, including the Saguambi, Mindo, and Canchupí rivers [6, 7]. The Saguambi River supplies 60% of fresh water and is impacted by soil removal, urbanization, and wastewater discharges [8]. The Mindo River, a popular tourist spot surrounded by diverse flora and fauna, is contaminated by chemicals, organic waste, soil erosion, and direct discharges from households [9]. The Canchupí River, used for bathing and laundry, crosses all urban areas of Mindo, and has higher anthropogenic contamination levels, including fecal bacteria and organic material from direct household discharges, due to the inefficiency of the wastewater treatment system [8].

Evaluating river contamination requires analyzing microorganisms such as fecal and environmental indicators (i.e., *Escherichia coli* and total coliforms), pathogen bacteria, and parasites. *E. coli*, originating mainly from human and animal intestines, promotes the presence of further primary and opportunistic pathogens such as *E. coli* pathotypes. Enteropathogenic *E. coli* (EPEC), enterohemorrhagic *E. coli* (EHEC), enteroinvasive *E. coli* (EIEC), and enteroaggregative *E. coli* (EAEC) are common in freshwater [1] and can cause diarrhea [10], with EHEC potentially leading to hemolytic uremic syndrome [11]. Parasites like *Giardia* and *Cryptosporidium* spp*.* cause zoonotic diseases [12] and spread through food and direct contact with fecal matter [13]. *Helicobacter pylori* causes gastrointestinal disorders and is linked to stomach cancer [14]. Other bacterial pathogens are *Mycobacterium tuberculosis* and *Mycobacterium leprae*, causing tuberculosis and leprosy [15–17], respectively, and are transmitted through person-to-person or fomites [18]. The increasing human population (ca. 4.500) and tourism in Mindo have led to heightened river pollution, urging microbiological analysis to determine water suitability for consumption and recreational activities.

## Main text

### Methods

#### Sample collection and preparation

**Fig. 1:**
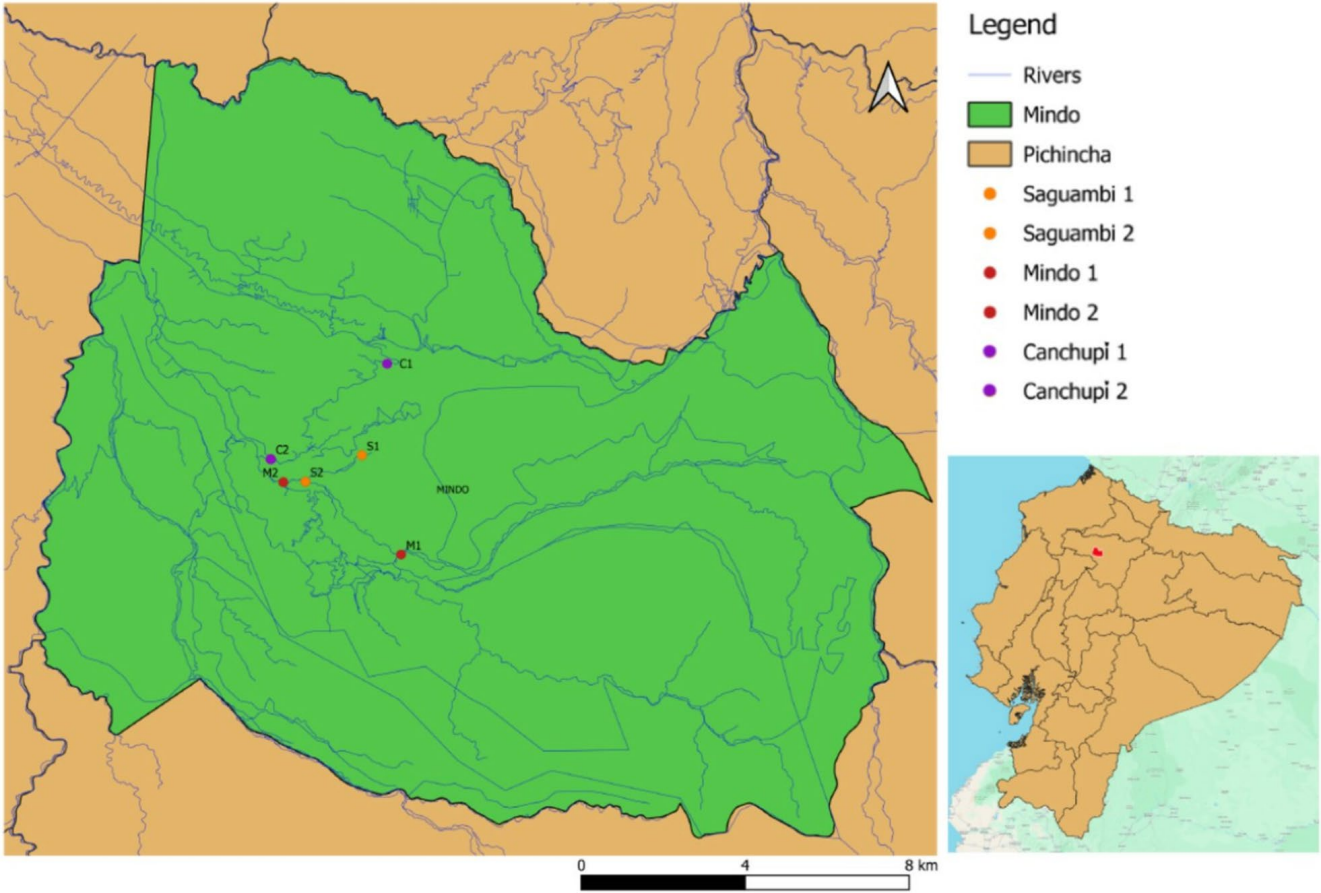
Geographical map of Mindo indicating the selected water sampling points across the Saguambi (S), Mindo (M), and Canchupí (C) Rivers: Point 1 represents the upstream location (before the Mindo community) and Point 2 represents the downstream location (after the Mindo community)

Water samples were collected from two points along each river: one upstream (before the community) and one downstream (after the community) of the human settlements. Before the community represents areas with minimal human impact, while after the community represents areas downstream, where anthropogenic activities are more pronounced. Therefore, water samples were collected from the Saguamby, Mindo, and Canchupí Rivers (see Additional file 1; Fig. 1), strictly following Borja et al. protocol [19]. Briefly, samples were collected by fully inverting the sample container and submerging it to a depth of 0.3 m below the water surface to avoid surface scum and debris. Glass containers, previously sterilized by autoclave at 121 °C for 15 min, were used for collection. A total volume of 800 mL was collected per river, and the samples were maintained at 4 °C during transport and processed within 6 h at the Institute of Microbiology at Universidad San Francisco de Quito (IM-USFQ). A total of 36 samples were analyzed: 12 per river, with duplicates taken at two points (before and after the community) across three seasons (dry, rainy, and transitional), resulting in 4 samples per river per season. No special permission was required in the present study to collect water samples as the water collection points are accessible to the public through any domestic and recreational activities. The filtration method was adapted from Dobrowsy et al. [20]. All samples destined for microbial analysis were filtered using a vacuum pump under aseptic conditions (Chemical Duty Pump, Millipore, Merck, Burlington, MA, USA) through a 0.45 µm nitrocellulose membrane (Millipore, Merck, Burlington, MA, USA) under aseptic conditions. The total volume of 800 mL of water was filtered per sample, and the membrane was placed in a Falcon tube with 20 mL of sterile distilled water. The tube was vortexed for 10–15 min at maximum speed to resuspend particles and microorganisms while preventing membrane breakage. After membrane removal, the tubes were centrifuged at 5000 rpm for 15 min, and the supernatant was discarded. The pellet was suspended in 2 mL of sterile distilled water and divided into three aliquots of 500 μL: one for DNA extraction, a second for bacterial growth cultures, and a third for storage.

### Cultivation of *E. coli* and total coliforms

For the quantification of *Escherichia coli* and total coliforms, an alternative method adapted from Pitkänen et al. [21] was employed. Successive tenfold dilutions of the initial aliquot were cultured in Chromocult agar medium (Biolab Laboratories, Merck Inc.) through the classic dilution technique. A volume of 100 µL from each dilution (10^⁻1^ to 10^-4^) was deposited in triplicate medium plates and incubated at 37 °C for 24–48 h. Results were recorded as colony-forming units (CFU) per milliliter of the original water sample (see Additional file 2).

### DNA extraction and molecular identification

Total environmental DNA was extracted using the DNeasy® PowerSoil® Pro kit (QIAGEN) for the analysis of all targeted pathogens, including bacteria and protozoa. Specific primers were used to identify bacterial genera/species through Polymerase Chain Reaction (PCR). One set of primers targeted the *16S rRNA* gene for general bacterial identification, while another set specifically targeted the *16S rRNA* gene for *Helicobacter pylori*. For the detection of *Escherichia coli* pathotypes, the following genes were targeted: *aggR* (EAEC), *stx* (EHEC), *eae* (EPEC), and *ipaH* (EIEC). Additionally, the *pra* gene was used to detect *Mycobacterium leprae*, the *MPB64* gene for *Mycobacterium tuberculosis*, the *COWP* gene for *Cryptosporidium* spp., and the *TPI* gene for *Giardia* spp. (see Additional File 3 for details on primers and PCR protocols). The PCR reaction was prepared in a final volume of 15 μL, with reagent amounts optimized based on the pathogen. Briefly, the final mix included: 3 μL of Green GoTaq® Flexi Buffer, 0.90–1.8 μL of 2.0 mM MgCl₂, 0.30–0.60 μL of 0.2 mM dNTP mix, 0.45–0.75 μL of each PCR primer, 0.08–0.10 μL of 0.5 U GoTaq® Flexi DNA Polymerase (Promega), 1–2 μL of DNA template, and DNA-free water to complete the final volume.

### PCR product analysis

PCR products were visualized using electrophoresis, with positive and negative controls provided by the IM-USFQ. All assays were performed in triplicate at different days. Positive PCR products were subjected to Sanger sequencing (Macrogen; Additional file 4).

## Results

### *Escherichia coli* and total coliform counts

**Fig. 2:**
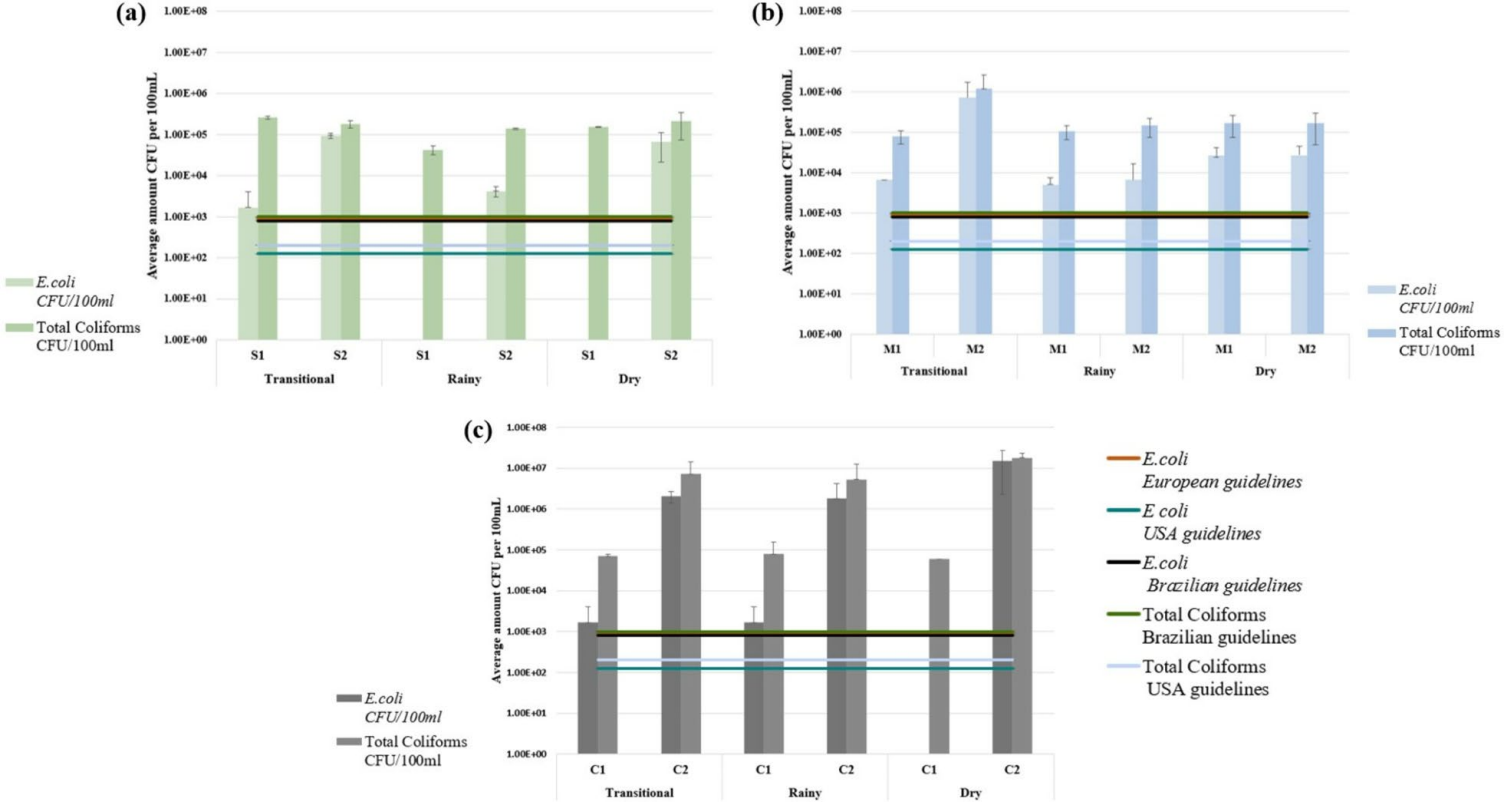
Average concentrations of *Escherichia coli* and total coliforms in **a** Saguambi River, **b** Mindo River, and **c** Canchupí River. Limit values are represented as follows: () *E. coli* ≤ 900 CFU/100 mL (European Union Directive 200/7/EC [25]), ( ) *E. coli* ≤ 126 CFU/100 mL; ( ) total coliforms ≤ 200 CFU/100 mL (U.S. EPA Recreational Water Quality Criteria [24]), and ( ) *E. coli* ≤ 800 CFU/100 mL; ( ) total coliforms ≤ 1000 CFU/100 mL (Brazilian Resolution CONOMA n°274 [26])

The study analyzed two collection points in the Saguambi, Mindo, and Canchupí Rivers during three seasons (see Fig. 1), demonstrating high concentrations of *E. coli* and total coliforms in all rivers, with most collection points exceeding international guidelines (see Fig. 2). The highest concentrations of *E. coli* and total coliforms were found in the Canchupí River during the dry season, with values of 1.50 × 10^7^ and 1.79 × 10^7^ CFU/100 mL, respectively. In the Saguambi River, the highest concentration of *E. coli* was detected after the community in the transitional season with 9.42 × 10^4^ CFU/100 mL, while the highest concentration of total coliforms was detected before the community at the same season, with a value of 2.58 × 10^5^ CFU/100 mL. Mindo River showed the highest values of both *E. coli* and total coliforms after the community sample collection point in the transitional season with 7.15 × 10^5^ and 5.85 × 10^5^ CFU/100 mL, respectively. The concentration of *E. coli* and total coliforms increased after the community, except for the Saguambi River, which showed higher values before the community in the transitional season.

### Molecular identification of pathogens

**Fig. 3:**
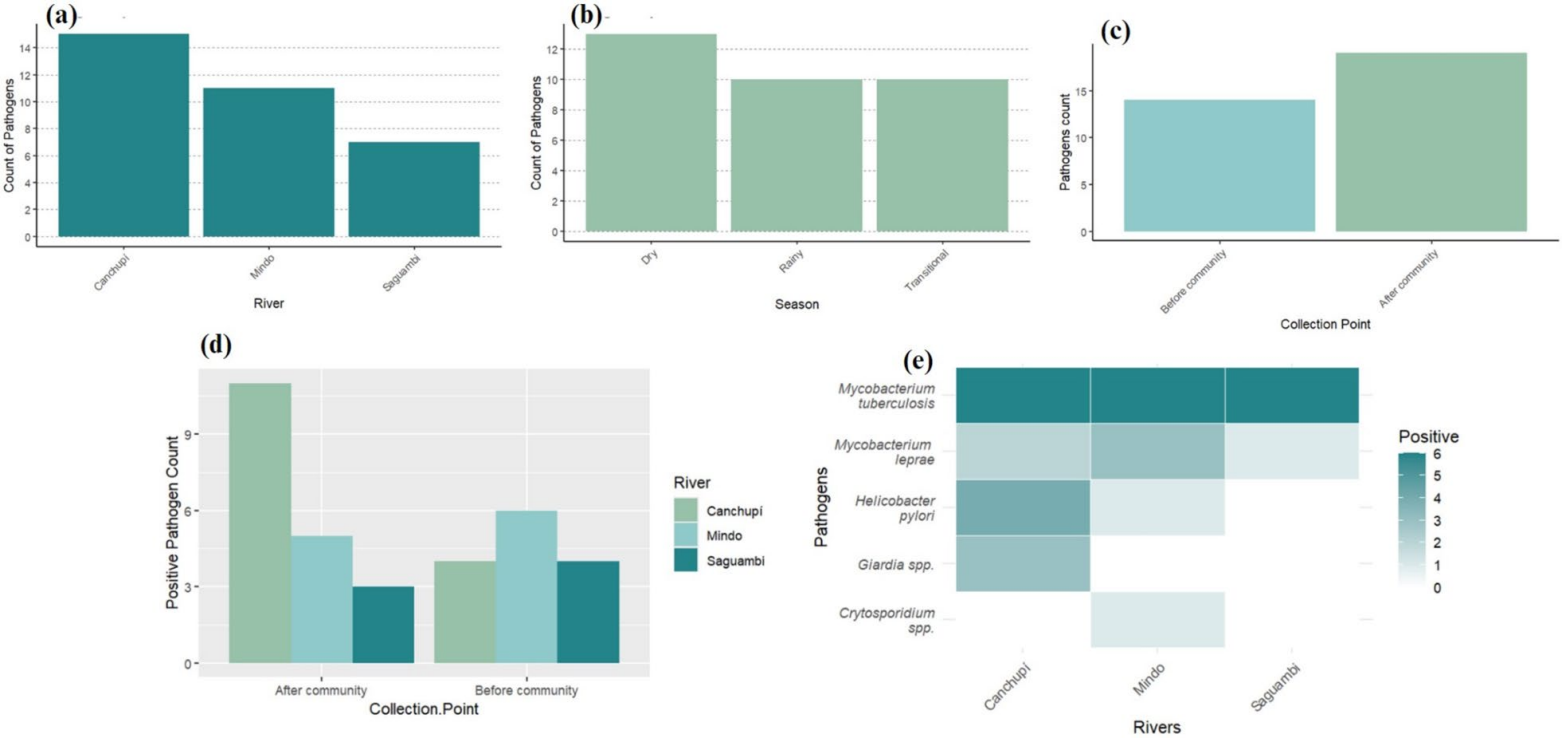
Detection of *Mycobacterium tuberculosis*, *Mycobacterium leprae*, *Helicobacter pylori*, *Giardia*, and *Cryptosporidium* spp. in the Saguambi, Mindo, and Canchupí Rivers. **a** Total pathogen counts across all three rivers. **b** Seasonal variation in pathogen counts. **c** Comparison of pathogen counts before and after the Mindo community. **d** Pathogen distribution by sampling points (before and after the community) for the Saguambi, Mindo, and Canchupí Rivers. **e** Heatmap illustrating pathogen counts in each river

Molecular identification via PCR detected the DNA of several pathogens in the three rivers, with Sanger sequencing (Macrogen, South Korea) confirming the initial positive results (Fig. 3). Clinically relevant pathogens were found, including *Mycobacterium tuberculosis* in all three rivers across all seasons*. Mycobacterium leprae* was present in the Saguambi River during the dry season, the Mindo River in all seasons, and the Canchupí River during transitional and dry seasons. *Helicobacter pylori* was identified in the Mindo River during the dry season and in the Canchupí River in all seasons. *Giardia* spp. was detected in the Canchupí River in all the seasons, while *Cryptosporidium* spp. was found in the Mindo River during the dry season. No *Escherichia coli* pathotypes were found in any of the rivers or seasons. The Canchupí River showed the highest pathogen levels after the community during the dry season. Mindo and Saguambi Rivers demonstrated a higher number of pathogens before the community. Sanger sequencing validated the initial molecular identification by PCR assays and identified some of the species of the detected pathogens (Additional file 4). For instance, selected *M. tuberculosis* samples showed 99.32% (T2 sample) and 98.33% (T3 sample) identity. Only one of the four selected samples (L1 sample) for DNA sequencing showed 98.57% identity for *M. leprae*. *H. pylori* was identified in two selected samples with 96.18% (P1 sample) and 87.95% (P4 sample) identity. One out of three *Giardia* samples was identified as *Giardia intestinalis* with 89.80% identity, but *Cryptosporidium* samples could not be identified by DNA Sanger sequencing.

## Discussion

In Mindo, water pollution threatens local health and tourism, a major income source. This biodiverse region, a bird conservation area, relies on its rivers for freshwater [22]. The Saguambi, Mindo, and Canchupí Rivers face significant contamination from wastewater discharges, with high levels of *E. coli*, total coliforms, and pathogens posing health risks [23].

Most collecting points across all seasons exceeded the permitted limit of *E. coli* and total coliforms set by the United States Protection Agency [24], European Union guidelines [25], and Brazilian guidelines [26]. All rivers showed higher *E. coli* and total coliform levels after the local community, except for total coliforms in the Saguambi River during the transitional season. A previous study on the Mindo River reported high levels of *E. coli* (1.72 × 10^1^ CFU/100 mL) and total coliforms (6.78 × 10^1^ CFU/100 mL) [19]. These levels significantly increased in 2023, with *E. coli* concentrations ranging from 5 × 10^3^ to 7.15 × 10^5^ CFU/100 mL and total coliforms from 8 × 10^4^ to 5.85 × 10^5^ CFU/100 mL. Similarly, the Canchupí River showed higher total coliform levels (6 × 10^4^ to 1.79 × 10^7^ CFU/100 mL) compared to previous reports (4.8 × 10^4^ CFU/100 mL) [2]. However, Castillo & Ullauri [2] employed a different methodology and collected samples only during November and December 2021. No comparable data exists for the Saguambi River, nor does previous research provide the detailed seasonal and locational data presented in this study for all three rivers.

Local and international studies found similar levels of *E. coli* and total coliforms exceeding international limits. Ecuadorian rivers such as Machángara, Guayllabamba, and Zamora showed high levels of *E. coli* and total coliforms (> 1.0 × 10^4^ CFU/100 mL), particularly near urban areas [19]. Similar contamination was found in Colombia, with the Salgar River registering 5.9 × 10^7^ *E. coli* [27], while the Choluteca River in Honduras showed total coliform levels of 6 × 10^4^ CFU/100 mL [28]. In Malaysia, the Larut River showed *E. coli* and total coliform levels of 4.1 × 10^5^ CFU/100 mL and 4.7 × 10^5^ CFU/100 mL [29], respectively. In India, the Kshipra River had lower levels of *E. coli* (5.2 × 10^3^ CFU/100 mL) and total coliforms (4 × 10^4^ CFU/100 mL), but still concerning levels [30]. These findings underscore the extensive impact of human activities, such as livestock, tourism, and urbanization, on river water quality, making it unsafe for consumption and recreation [31].

The study identified the genetic material of numerous clinically relevant pathogens in all the rivers. While this finding highlights potential public health concerns, it does not confirm the presence of viable organisms or immediate health risks (see Fig. 3). *M. tuberculosis* DNA was detected in all rivers and seasons, a concerning finding due to its environmental persistence and potential spread among livestock and wildlife. Further studies are needed to confirm these findings and trace the contamination source, possibly wild animals [12]. Similar findings have been reported in water sources in southern Spain [32], rivers near hospital areas in Nigeria [33], and the Yellow River in China [34]. Literature suggests that *M. tuberculosis* can survive in rivers for up to 50 days at temperatures of 8 to 20 ºC [35].

*Mycobacterium leprae* DNA was found in the Saguambi River during the dry season, the Mindo River in all seasons, and the Canchupí River during transitional and dry seasons. Untreated leprosy patients and animal reservoirs like armadillos are known infection sources [36]. Research in Brazil, India, Indonesia, Surinam, England, and Bangladesh has shown that *M. leprae* can survive outside the human body for up to 46 days in moist soil, potentially becoming a source of infection. Further studies are needed to understand the transmission modes [37].

*Helicobacter pylori* DNA was detected in the Mindo River during the dry season and in the Canchupí River throughout all seasons. This significant pathogen, associated with gastric infections and cancer [14], has also been identified in freshwater sources worldwide, including the Bogotá River in Colombia [38] and the Nairobi River basin in Kenya [39]. Although direct links between waterborne *H. pylori* and gastric cancer are limited, its detection in water highlights the need for quality monitoring and effective treatment to reduce transmission and related gastric disease risks [40].

The detection of *Giardia intestinalis* and *Cryptosporidium* spp. DNA in the Canchupí and Mindo Rivers, respectively, indicates potential health risks for adjacent communities, as these protozoa can cause gastrointestinal illnesses, particularly in children [41, 42]. The elevated levels of *E. coli* and total coliforms in the Canchupí River further suggest fecal contamination, likely from livestock, agriculture, tourism, soil erosion, urbanization, and inadequate wastewater treatment.

Studies have demonstrated that microbial contamination in rivers is closely linked to land use patterns and environmental factors [31, 43, 44]. For instance, research on the Umhlangane River in Durban, South Africa, found that microbial indicators varied with environmental conditions, affecting water treatment processes and public health outcomes [45]. Similarly, a study on the Mat River in Albania highlighted the use of bacteria and benthic macroinvertebrates as indicators for water quality assessment, emphasizing the need for comprehensive monitoring strategies [46]. To mitigate contamination in these rivers, it is essential to implement effective water quality monitoring and management strategies. Eco-engineered approaches, such as constructed wetlands and riparian buffer zones, have been shown to remediate polluted rivers by reducing pollutant loads and improving water quality [47]. Additionally, public health interventions, including community education on sanitation practices and the development of adequate wastewater treatment facilities, are crucial steps toward reducing the transmission of waterborne pathogens and safeguarding community health.

In conclusion, the findings of this study underscore the need for integrated water resource management and public health strategies to address microbial contamination in the Mindo region's rivers. By drawing on associations between microbiological parameters and environmental factors, as demonstrated in global studies, targeted interventions can be developed to mitigate health risks and improve water quality for the affected communities.

### Limitations

This study has several limitations: (1) physicochemical parameters and antibiotic resistance were not evaluated; (2) the analysis was limited to detecting DNA, without confirming the presence of living organisms; and (3) other types of pathogens were not included in the evaluation.

## Supplementary Information

Additional file 1: Table S1. General information on sample collection from the Saguambi, Mindo, and Canchupí Rivers. Sampling points: (1) upstream (before the community); (2) downstream (after the community). Table S2. Average concentrations of *Escherichia coli* and total coliforms in the Saguambi, Mindo, and Canchupí rivers across three seasons. (a) Concentrations of *Escherichia coli* and total coliforms as CFU/100 mL (colony-forming unit per 100 mL). (b) Average concentrations measured as CFU/100 mL, including duplicate samples. (c) SD: Standard deviation values. Table S3. Primers and PCR cycling parameters for detecting potential pathogens. Table S4. Sanger sequencing results for pathogen identification. (a) Consensus sequences represent the correct order of a sequence. (b) Percentage of identity with other sequences in BLASTn (https://blast.ncbi.nlm.nih.gov/).

## Data Availability

Data is provided within the manuscript or supplementary information files.

## Abbreviations

CFU: Colony-forming units
EPEC: Enteropathogenic* E. coli*
EHEC: Enterohemorrhagic *E. coli*
EIEC: Enteroinvasive* E. coli*
EAEC: Enteroagregative *E. coli*
PCR: Polymerase chain reaction
*H. pylori*: *Helicobacter pylori*
*M. tuberculosis*: *Mycobcaterium tuberculosis*
*M. leprae*: *Mycobacterium leprae*
