# Supplementary material for: Pilot study on seasonal variability in microbial contamination in the developing tourist region of Mindo, Ecuador: a comparative analysis of the Saguambi, Mindo, and Canchupí Rivers
Source: BMC Res Notes. 2025 Apr 10;18:156. doi: 10.1186/s13104-025-07205-3 (PMC11983951; doi:10.1186/s13104-025-07205-3)
Supplement: Supplementary file 1 — Additional file 1: Table S1. General information on sample collection from the Saguambi, Mindo, and Canchupí Rivers. Sampling points: (1) upstream (before the community); (2) downstream (after the community). Table S2. Average concentrations of Escherichia coli and total coliforms in the Saguambi, Mindo, and Canchupí rivers across three seasons. (a) Concentrations of Escherichia coli and total coliforms as CFU/100 mL (colony-forming unit per 100 mL). (b) Average concentrations measured as CFU/100 mL, including duplicate samples. (c) SD: Standard deviation values. Table S3. Primers and PCR cycling parameters for detecting potential pathogens. Table S4. Sanger sequencing results for pathogen identification. (a) Consensus sequences represent the correct order of a sequence. (b) Percentage of identity with other sequences in BLASTn (https://blast.ncbi.nlm.nih.gov/). [file 13104_2025_7205_MOESM1_ESM.docx]

**Additional file 1: Table S1.** General information on sample collection from the Saguambi, Mindo, and Canchupí Rivers.

| **River** | **GPS Coordinates** | **Season** | **Date of Collection sampling** |
| --- | --- | --- | --- |
| Saguambi | (1)  0°3'8" S 78°45'47" W  (2) 0°03 28.6"S 78°46'31.6"W | Transitional | 2/4/2023 6/25/2023 |
|  |  | Rainy | 4/2/2023 5/27/2023 |
|  |  | Dry | 8/26/2023 9/23/2023 |
| Mindo | (1) 0°4'26" S 78°45'16" W (2) 0°3'29" S 78°46'49" W | Transitional | 2/4/2023 6/25/2023 |
|  |  | Rainy | 4/2/2023 5/27/2023 |
|  |  | Dry | 8/26/2023 9/23/2023 |
| Canchupí | (1) 0°2'3" S 78°45'21" W (2) 0°3'11" S 78°46'59" W | Transitional | 2/4/2023 6/25/2023 |
|  |  | Rainy | 4/2/2023 5/27/2023 |
|  |  | Dry | 8/26/2023 9/23/2023 |

Legend: Sampling points: (1) upstream (before the community); (2) downstream (after the community).

**Additional file 2: Table S2.** Average concentrations of *Escherichia coli* and total coliforms in the Saguambi, Mindo, and Canchupí rivers across three seasons.

|  | | | | ***Escherichia coli*** | | | **Total coliforms** | | |
| --- | --- | --- | --- | --- | --- | --- | --- | --- | --- |
| **River** | **Season** | **Collection** | **Collection** | **CFU/100ml^(a)^** | **Average amount** | **SD^(c)^** | **CFU/100ml^(a)^** | **Average amount CFU/100mL^(b)^** | **SD^(c)^** |
| **Saguambi** | **Transitional** | Before the community | S1. T1 | 3.33E+03 | 1.67E+03 | 2.36E+03 | 2.43E+05 | 2.58E+05 | 2.12E+04 |
|  |  | Before the community | S1. T2 | 0.00E+00 |  |  | 2.73E+05 |  |  |
|  |  | After the community | S2. T1 | 8.50E+04 | 9.42E+04 | 1.30E+04 | 2.10E+05 | 1.82E+05 | 4.01E+04 |
|  |  | After the community | S2. T2 | 1.03E+05 |  |  | 1.53E+05 |  |  |
|  | **Rainy** | Before the community | S1. R1 | 0.00E+00 | 0.00E+00 | 0.00E+00 | 5.00E+04 | 4.25E+04 | 1.06E+04 |
|  |  | Before the community | S1. R2 | 0.00E+00 |  |  | 3.50E+04 |  |  |
|  |  | After the community | S2. R1 | 5.00E+03 | 4.17E+03 | 1.18E+03 | 1.37E+05 | 1.40E+05 | 4.71E+03 |
|  |  | After the community | S2. R2 | 3.33E+03 |  |  | 1.43E+05 |  |  |
|  | **Dry** | Before the community | S1. D1 | 0.00E+00 | 0.00E+00 | 0.00E+00 | 1.53E+05 | 1.52E+05 | 2.36E+03 |
|  |  | Before the community | S1. D2 | 0.00E+00 |  |  | 1.50E+05 |  |  |
|  |  | After the community | S2. D1 | 1.00E+05 | 6.75E+04 | 4.60E+04 | 3.05E+05 | 2.10E+05 | 1.34E+05 |
|  |  | After the community | S2. D2 | 3.50E+04 |  |  | 1.15E+05 |  |  |
| **Mindo** | **Transitional** | Before the community | M1. T1 | 6.67E+03 | 6.67E+03 | 0.00E+00 | 6.00E+04 | 8.00E+04 | 2.83E+04 |
|  |  | Before the community | M1. T2 | 6.67E+03 |  |  | 1.00E+05 |  |  |
|  |  | After the community | M2. T1 | 1.40E+06 | 7.15E+05 | 9.69E+05 | 1.00E+06 | 5.85E+05 | 5.87E+05 |
|  |  | After the community | M2. T2 | 3.00E+04 |  |  | 1.70E+05 |  |  |
|  | **Rainy** | Before the community | M1. R1 | 3.33E+03 | 5.00E+03 | 2.36E+03 | 7.67E+04 | 1.06E+05 | 4.12E+04 |
|  |  | Before the community | M1. R2 | 6.67E+03 |  |  | 1.35E+05 |  |  |
|  |  | After the community | M2. R1 | 0.00E+00 | 6.67E+03 | 9.43E+03 | 9.50E+04 | 1.48E+05 | 7.42E+04 |
|  |  | After the community | M2. R2 | 1.33E+04 |  |  | 2.00E+05 |  |  |
|  | **Dry** | Before the community | M1. D1 | 1.67E+04 | 2.67E+04 | 1.41E+04 | 1.00E+05 | 1.65E+05 | 9.19E+04 |
|  |  | Before the community | M1. D2 | 3.67E+04 |  |  | 2.30E+05 |  |  |
|  |  | After the community | M2. D1 | 1.33E+04 | 2.67E+04 | 1.89E+04 | 8.33E+04 | 1.69E+05 | 1.21E+05 |
|  |  | After the community | M2. D2 | 4.00E+04 |  |  | 2.55E+05 |  |  |
| **Canchupí** | **Transitional** | Before the community | C1. T1 | 0.00E+00 | 1.67E+03 | 2.36E+03 | 6.50E+04 | 7.00E+04 | 7.07E+03 |
|  |  | Before the community | C1. T2 | 3.33E+03 |  |  | 7.50E+04 |  |  |
|  |  | After the community | C2. T1 | 1.60E+06 | 2.05E+06 | 6.36E+05 | 2.40E+06 | 7.20E+06 | 6.79E+06 |
|  |  | After the community | C2. T2 | 2.50E+06 |  |  | 1.20E+07 |  |  |
|  | **Rainy** | Before the community | C1. R1 | 3.33E+03 | 1.67E+03 | 2.36E+03 | 2.50E+04 | 8.00E+04 | 7.78E+04 |
|  |  | Before the community | C1. R2 | 0.00E+00 |  |  | 1.35E+05 |  |  |
|  |  | After the community | C2. R1 | 1.25E+05 | 1.81E+06 | 2.39E+06 | 1.40E+05 | 5.32E+06 | 7.33E+06 |
|  |  | After the community | C2. R2 | 3.50E+06 |  |  | 1.05E+07 |  |  |
|  | **Dry** | Before the community | C1. D1 | 0.00E+00 | 0.00E+00 | 0.00E+00 | 6.00E+04 | 6.00E+04 | 0.00E+00 |
|  |  | Before the community | C1. D2 | 0.00E+00 |  |  | 6.00E+04 |  |  |
|  |  | After the community | C2. D1 | 6.00E+06 | 1.50E+07 | 1.27E+07 | 2.15E+07 | 1.79E+07 | 5.07E+06 |
|  |  | After the community | C2. D2 | 2.40E+07 |  |  | 1.43E+07 |  |  |

Legend: (a) Concentrations of *Escherichia coli* and total coliforms as CFU/100mL (colony-forming unit per 100 mL). (b) Average concentrations measured as CFU/100 mL, including duplicate samples. (c) SD: Standard deviation values.

**Additional file 3: Table S3.** Primers and PCR cycling parameters for detecting potential pathogens.

| **Organism** | **Primer name** | **Primer sequence (5′–3′)** | **PCR mixture** | **PCR cycling parameters** | **Gene (size [bp])** | **References** |
| --- | --- | --- | --- | --- | --- | --- |
| ***Single PCR assays*** | | | | | | |
| Universal | Forward: fDD2 | CCGGATCCGTCGACAGAGTTTGATCITGGCTCAG | 3 μl of Green GoTaq Flexi Buffer, 1.2μl of 2.0 mM MgCl_2, 0.30 μl of 0.2 mM dNTPs mix, 0.75 μl for each PCR primer, 0.10 μl of 0.5U Go Taq Flexi DNA polymerase, 2 μl of DNA. | 3 min at 94°C; 35 cycles of 94°C for 30 s, 54°C for 30 s, 72°C for 1.5 min | *16S* rRNA (1,600) | (Dobrowsky et al., 2014) |
|  | Reverse: rPP2 | CCAAGCTTCTAGACGGITACCTTGTTACGACTT |  |  |  |  |
| *Helicobacter pylori* | Forward: | GCGGGATAGTCAGTCAGGTG | 1.5 μl of 2.0 mM MgCl_2, 0.38 μl of 0.2 mM dNTPs mix, 0.75 μl for each PCR primer, 0.10 μl of 0.5U Go Taq Flexi DNA polymerase, 2 μl of DNA | 2 min at 94°C; 40 cycles of 94°C for 30 s, 60°C for 30 s, 72°C for 1 min | *16S* rRNA (706) | (Valenzuela & Machado, 2016) |
|  | Reverse: | AAGATTGGCTCCACTTCGCA |  |  |  |  |
| EAEC | Forward: AggRKs1 | GTATACACAAAAGAAGGAAGC | 3 μl of Green GoTaq Flexi Buffer, 1.2 μl of 2.0 mM MgCl_2, 0.30 μl of 0.2 mM dNTPs mix, 0.75 μl for each PCR primer, 0.10 μl of 0.5U Go Taq Flexi DNA polymerase, 2 μl of DNA | 2 min at 95°C; 35 cycles of 95°C for 1 min, 54°C for 1 min, 72°C for 1 min | *aggR* (254) | (Ramírez Castillo, Avelar González, Garneau, Díaz, et al., 2013) |
|  | Reverse: AggRkas2 | ACAGAATCGTCAGCATCAGC |  |  |  |  |
| EHEC | Forward: VTcomU | GAGCGAAATAATTTATATGTG |  |  | *stx* (518) |  |
|  | Reverse: Vtcomd | TGATGATGGCAATTCAGTAT |  |  |  |  |
| EPEC | Forward: SK1 | CCCGAATTCGGCACAAGCATAAGC |  |  | *eae* (881) |  |
|  | Reverse: SK2 | CCCGGATCCGTCTCGCCAGTATTCG |  |  |  |  |
| EIEC | Forward: IpaIII | GTTCCTTGACCGCCTTTCCGATACCGTC |  |  | *ipaH* (619) |  |
|  | Reverse: IpaIV | GCCGGTCAGCCACCCTCTGAGAGTAC |  |  |  |  |
| *Mycobacterium leprae* | Forward: S13 | CTCCACCTGGACCGGCGAT | 0.90 μl of 2.0 mM MgCl_2, 0.30 μl of 0.2 mM dNTPs mix, 0.60 μl for each PCR primer, 0.10 μl of 0.5U Go Taq Flexi DNA polymerase, 2 μl of DNA | 5 min at 95°C; 30 cycles of 94°C for 2 min, 58°C for 1 min, 72°C for 2min | *pra* (531) | (Arunagiri, Sangeetha, Sugashini, Balaraman, & Showkath Ali, 2017) |
|  | Reverse: S62 | GACTAGCCTGCCAAGTCG |  |  |  |  |
| ***Nested PCR assays*** | | | | | | |
| *Mycobacterium tuberculosis* | Forward: Mpb1 | TCCGCTGCCAGTCGTCTTCC | 0.90 μl of 2.0 mM MgCl_2, 0.30 μl of 0.2 mM dNTPs mix , 0.50 μl for each PCR primer, 0.10 μl of 0.5U Go Taq Flexi DNA polymerase, 2 μl of DNA | 5 min at 95°C; 30 cycles of 95°C for 30 s, 54°C for 30 s, 72°C for 30 s | *MPB64* (240) | (Madhavan, Therese, Gunisha, Jayanthi, & Biswas, 2000) |
|  | Reverse: Mpb2 | GTCCTCGCGAGTCTAGGCCA |  |  |  |  |
|  | Forward: Mpb3 | ATTGTGCAAGGTGAACTGAG |  | 5 min at 95°C; 35cycles of 95°C for 30 s, 58°C for 30 s, 72°C for 30 s | *MPB64* (200) |  |
|  | Reverse: Mpb4 | AGCATCGAGTCGATCGCGGA |  |  |  |  |
| *Cryptosporidium* spp. | Forward: Cry 15 | GTAGATAATGGAAGAGATTGTG | 1.8 μl of 2.0 mM MgCl_2_, 0.60 μl of 0.2 mM dNTPs mix, 0.45 μl for each PCR primer, 0.10 μl of 0.5U Go Taq Flexi DNA polymerase, 1 μl of DNA | 10 min at 95°C; 45 cycles of 94°C for 30 seconds, 52°C for 30 seconds, 72°C for 50 seconds | *COWP* (550) | (Salza, 2014; Yu, Lee, & Park, 2009) |
|  | Reverse: Cry 9 | GGACTGAAATACAGGCATTATCTT |  |  |  |  |
|  | Forward: Cowpnest F | TGTGTTCAATCAGACACAGC | 1.8 μl of 2.0 mM MgCl_2_, 0.60 μl of 0.2 mM dNTPs mix, 0.45 μl for each PCR primer, 0.10 μl of 0.8U Go Taq Flexi DNA polymerase, 1 μl of DNA | 10 min at 95°C; 32 cycles of 94°C for 30 seconds, 60°C for 30 seconds, 72°C for 50 s. | *COWP* (311) |  |
|  | Reverse: Cowpnest R | TCTGTATATCCTGGTGGG |  |  |  |  |
| *Giardia* spp. | Forward: AL3543 | AAATTATGCCTGCTCGTCG | 1.8 μl of 2.0 mM MgCl_2_, 0.60 μl of 0.2 mM dNTPs mix, 0.45 μl for each PCR primer, 0.10 μl of 0.5U Go Taq Flexi DNA polymerase, 1 μl of DNA | 5 min at 94°C; 35 cycles of 94°C for 45s, 50°C for 45 s, 72°C for 1 min | *TPI* (605) | (Salza, 2014) |
|  | Reverse: AL3546 | CAAACCTTTTCCGCAAACC |  |  |  |  |
|  | Forward: AL3544 | CCCTTCATCGGTGGTAACTT | 1.8 μl of 2.0 mM MgCl_2_, 0.60 μl of 0.2 mM dNTPs mix, 0.45 μl for each PCR primer, 0.10 μl of 0.8U Go Taq Flexi DNA polymerase, 1 μl of DNA | 5 min at 94°C; 35 cycles of 94°C for 45s, 53°C for 30 s, 72°C for 1 min | *TPI* (530) |  |
|  | Reverse: AL3545 | GTGGCCACCACTCCCGTGCC |  |  |  |  |

**Additional file 4: Table S4**. Sanger sequencing results for pathogen identification.

| **Sample** | **Collection time** | **Consensus sequence (a)** | **Result** | **Identity** | **GenBank accession numbers (Release Date: 20 August 2025)** |
| --- | --- | --- | --- | --- | --- |
| T2 | C1. S1 | TGGGACCAATACCTGGGTTGGGCCGGCTGCTTCGGGCAGCAACTCCCCCGGGTTGAAGAAGAAAATCACCCCGTCGTTCGTGACTGCGAAGTTCT  GATAATTCACCGGGTCCTAGCCGGCATTCGGCGCTATCGATACCTGTTGTCCG | *Mycobacterium tuberculosis* | 99.32 | PQ223724 |
| T3 | S1. T1 | CGCCGAATGCCGGCTTTGGACCCGGTGAATTATCAGAACTCGCAGTCACGAACGACGGGGTGATTTTCTTCTTCAACCCGGGGGAGTTGCTGCCC  GAAGCAGCCGGCCCAA*TCCCA***GGT | *Mycobacterium tuberculosis* | 98.33 | PQ223725 |
| L1 | M1. T2 | GAGGCCTACACATTCTGGGTCACCCGGATGCTGGCTTATGTCATCGACAACATCTCAGCCACGGTCCTGCTCGGCATTGGCATGTTGATTCACACG  CTCACGAAGCAAGAGGCGTGCGTCACTGATATCACGCAGTACAATGTTA*TCAGTACTGTGCTACTCAGCCTACCGGCATCGGCATGTTGGCGTTC  TGGTTCGCATGGTTGATGGCGACGGCCTACCTGGTCTGGAACTACGGCTATCGCCAGGGCGCCACCGGCTCCAGCATTGGCAAGACGGTAATGAA  GTTCAAGGTGATCAGCGAGGCTACTGGGCAGCCAATCGGTTTCGGTATGTCGGTGGAGCGCCA | *Mycobacterium*  *leprae* | 98.57 | PQ223726 |
| L3 | M2. S2 | C*TGTTGGTGTGCA*CGCCATCTACTCCGGGACGT*GCCGGGCGGTTCTAGAACTGTCAAGTCAT*GACTGTGCATGA*CCTA**CA**CCAGCCGG  GGGATGCACGCGTTGATCGACCGGGCGTTTTATCTGCCGAAGTCCTGGACCGATGACCCGGCACGCTG**CGCC**GAGGCCGGCGTGCCCGCCG  GGATCGAGTTCGCGACCAAACCCGCCCTGCTCACAGCGATGATCCTCGGCGCGTTCGATGCCGCATTCCCGTTCGGGTGGGTGGCCGGCGACGA  GGTGTACGGCGCGGACCCCGATCTGCAAACAACCCTGCAGGACAGGCAAATCGGCTACGTGCTGGCGATCGTTTGCGACCGACG**GGATAACC  ACCGAGGCGGGTC*ACACCCGGTCAGCGATCTGGCCGCCG | - | - | - |
| L4 | M1. L2 | ACTAGCCCCGCAAGTCGGATAGTCCGCTGCCGGTAGTGATCCTGAGATATTTTTCGATTCGACATCCGCTGGCGATTGTGACCATAAAACGGCCCT  CGCACTCACCATGCTCCTTGCA*GAGGTTCAGCCC*GCGACGGGCCTCTCGCTTTTGGTT*TATTCGCATGTCGATTCCC*TGGAAGCAACCGACCT  TGAGGTCGAGAGCGATCATCTTCTGAGTATGGGTCCTTGGAGACCCTTTTCCCGCAGATTTCGCGCCCAAATCGCTTGACGGCTCAGAGGAGACG  CAAATCGAAGTGCGCTCCCCCTGCCATCCGAAAGATTTCGATTCGCCGGCATGACTTG*ACCTGAAATTCCCAGCGCC*GGAAAAT*ATGGCCGGC  AGCAAAC*GAAGAAGTCATACCCAACGCCGGTTTCAGAGCAAGTCGAGGAGCTGACATTCTCCCAGTCGTCCCGCGA | - | - | - |
| L5 | C2. S1 | ACTCCGGCTACGCTCATTTCATCTCGAAAACTGCCCCCTCGGTCCTGGTCTGCATTACCATCTCCGTCCGGACCCTCTCTTGATCAACCGGGCGTGT  CATCTATCTCACTCCTGGAATATAATCGGAACGGTCCTCCACTCAGGCCGGCGTGCATCGGC**AGTCTTC**GAGGCTCTGGCT*ACACGATTCTGG  TTAAGCCCTAATCCTAGACGGGCTCGAGTCATCGTCACGATCCGAGCCAA**TCGGCTGGCTCG*GCGTTACCAAGACGCATGTTGA***AGA*CCC  GGTC*ACGAAGCTAGAGGCGTGCGGCAC*CGCATGTCAGGCAGTACAATGCTGACCATCAGCTGTGCGA*CGCGGAATATCC*TGCCAGG*GCATC  TGCATCTG*CGCT*TTGTGGC*CGCCTGGACGT*ACGGAGAAAGACCTGGACCGGCAGAAAACTAGCAGAGATC**ATGGGGGACGAAACTCGAC  CAGTCTCCAGCCGG*ATCTGACAGGGTGCCACAACTTGCGGGGCTAGTCCAAAAAAAAAAAAAAAAAAAAAAAGTGTCCTTGGTATGCAATTGG  TTCGGCGATGTGTGCGGAGCGGACCCCGGGGCACTAACAC | - | - | - |
| P1 | C1. L2 | TGACTGACTATCCCGGCCTGACTGGTATCCAGCGGCC**GACTGACTATCCCGTCGGTCCCCCCCTTCCTCCGCATTACGGAGGCAGGACATCGGG  AGAGTTCTCAG*CATGACCTGTTAGCAACTAAGAAAGGGGGTTGCGCTCGGTTGCGGGGCTTAACCCAAACATCTCACGACATGAGCTGACGAC  AGCCGTGCAGCACCTGTTTTCAAGGTCTAGCAAGCTAGA*CACTCCACTATTTCTAGCGGATTCTCTCAATGTCAAGCCTAGGTAA**GGTTCTTCG  *TGTATCTTCGAATTAAACCAC***ATGCTCC**ACCGCTTGTGCGGTTCCCCGTCTATTCCTTTGAGTTTTAATCTTGCGACCGTACTCCCCAGGGCG  GGATGCTTAATGCGTTAGCGGGTTTACTGGAGAGACTAAGCCCTCCAACAACTAGCATCCATCGTTTAGGGCG*****GGACTACCAGGGTATCTAAT  CCTGTTAGCTCCCCACGCTTTCGCGCAATCAGCGTCAGTAATGTTC*CAGCAGGTCGCCTTCGCAATGAGTATTCCTCTTGATCTCTACGGATTT**TA  CC****CCTAC****** AC****** | *Helicobacter*  *pylori* | 96.18 | PQ202268 |
| P4 | C2. S2 | AACTAGTTCGGTG*T*TAGTCGAGATCGCATCTTGATGACTT***GTCGTAGGTCCCACCTTACTC*CTCATTACGGAAACAGTATCCTTAGAG*TTCTC  AGCATGACCTGTTAGCCACTAAGAAAGGGGGCTGCGTTCGTTGTATCA*GGCTTAACCCAACCTCTAACGATTCGA*GTGCTGACAACAGCCGTGTT  GAAGCCCATGTTTGC*AAGGACTAACAAGCTAGACGCTCCACTATTTCTAGCGGATTTTCTCAATGGCTAACCTAGGCAAGGTTCTTCGTGTATCTTC  GAATTAAACCACATGCTCCACCG**CTTG*TGCGGACCC*CGTCTATTCCTTTGAGTTTTAATCTTGCGACCGTACTCCCCAGGCGGTATGCTTAATGC  GATAGATGCATTACTGGAGAGACT**AAGACCTCCAACAACTAGCATTGATCGTTTACGTCGTGTCCTACCAGCCCA**TCTGATCCTGTTTGCTCCC  CACGCTTTCCCGCAATCAGCGACAGTAACGTCCCAGCCAGTGGCCTTCCCAATGAGTACTCTCTCTTGATCTCTACGGATTTGCCCCTACACCAGG  AATTCCACCTACCTATTCCACACTCTAGA*TAGTAGAT*CAA*ATGC | *Helicobacter*  *pylori* | 87.95 | PQ202269 |
| A1 | C2. T2 | AGGGGAATTTTTTTTGATCATACTTTTTTTCTTGAACTAGTGTCTTAAGGCCCGCCTGGCCTTTCCGTCTGCGAAATCGGGCAAGCTCG*AAAACGG  AATAATTCAG*ATGCTTGTGCAAGCTAGGTGAGTCGTGTGCATT*CTATTCTCCGAGATTCTCGACTCATGCTAGGTGTTTTGGCCA*CCTAATCCGG  GCTTATGTCTTTTTGGATTGGTGAAA*TGAAC*GAATTGGGTGAAAA*TAG*CGGCGCTT*TAGGATTG*CGTCGCGGGAATTGTTGATTGGAAGGT  TGACC*CGAGTGTTGAGATGCT*TCTGGACCTGGGCCTCACCCAACCTGCGGCCCCG*TAATAAAACGCCAACCCCCCACGAGAGCACCGGTACA  AAAACGGGGGAGCAAACCAACGAGCAGAGAGCCCAAGTAGCCTAAGCGAGCTCTGGAGAAGGGCATGATGGTCATCTTCTGCACTGGGGAGAC  ACTGGACGAGCGCAAGGCCGACAAGACTATGGATGTGAACATTGCACAGCTCGAGGTCCTTAAAAAGAAACCCGATATCGATAAATGCTCTGGAA  GAGTGTCCTCATCGCCCTACGA | *Giardia*  *intestinalis* | 89.8 | PQ223727 |
| A2 | C2. S2 | CGACTGAACTTGAATTGAGGGGTTTAGCCAGGAGGGGACGTCTCGAAGGTTTCTTAGAGACGATTCAGCTGGGCGCACACCTGAATTTTATTCAA  CATTCGCAGCTCTTCGGAAGCCGCCAAATGCTCTCAATTA*******TCCCTTCTACCTTCCGGATCTTTATCTTACTGTGGC*AAGTTCCGGGGGTTT  CTTGTCACCGT*G**CC*AG*TGG*T*TGCCCCCGA**GAGCAGCGGGAACCAACGTCGCGACGACGTGCTT****CTGCCGAATTCGCAGGAGGTC  GTACAAGGTGGCCTGGATTGCTTCCAGAACCGTCTTCATCGCTACGGCGGATTGCTGGTGCCCACA**CGTGCCGCGTCGACAAGAAGTCGCCCC  CCAAACAAAATCCGCGACCATGTGCGA*TTC*GCCTTTACCATCTGCAGCAG*GTCTTGCCGAAGCCT*GCTGTCCTAGCGAAAGC*AGATCAAGC  ACCGGCGATAGGCGAAAGGGACTCCCGGGTTGTGCACAAATTTTTCCCCCCTTTAAATCACGATCCTGAAGGGAGGAGGGATCATCTAGAGCATC  AACGGTTTCCCAAGACCAGGAAAGTAAAAAGAAATAACGAGTGAACTAGACATGAATCGTATCTGACAAAACTCCGAACCAAGGGGACTCCAAT  TAGCCTAAACTGAAAGTTAACC | - | - | - |
| A3 | C2. L2 | CGTAGATTCCCCACTCTGGGGATGAGATTGTTGAGTCCTGGTGTGGAGAGTGTGTGCGGGCATCATTCCGAACCCTTATCTTTTCTTGCCGACAATT  TCAGAGCGCCCTAGAC*TGTACACT*TCCCAAACCATAGTG*TGTGGAATGATGCAAGTCTATCATTCCT*TCTAGAACATGGTCCGGAGTCGGGAAT  TGTCGTTGACCACTGAAGGTACC***CCCGTCATACCTACTCCGGCCGCCAACTGGATGGATTG**GCTAACCTGTACGTCATCCGAGTCCCTCATGG  GAGGGCCCGTCTACAGAACCATCGCATAGACACCTATG*GAGCCT***TTCC**CAGAAGTCTGTGATGTGAAGACATACATGT*CT*G*GGCTGTTAA  AAAGGGACGGAAAAAACGAAAAAAAAAAAAAAAAAAAAAAAAAAATGCTCGCCAGTCATCCCGCTCAACACCCTCCCCGACGGGC  TTCCGCCATCCCCCCCCAAGGGGGGGGAACCAGGGAG | - | - | - |
| C1 | M1. S2 | CGTAGATTCCCCACTCTGGGGATGAGATTGTTGAGTCCTGGTGTGGAGAGTGTGTGCGGGCATCATTCCGAACCCTTATCTTTTCTTGCCGACAAT  TTCAGAGCGCCCTAGAC*TGTACACT*TCCCAAACCATAGTG*TGTGGAATGATGCAAGTCTATCATTCCT*TCTAGAACATGGTCCGGAGTCGGGA  ATTGTCGTTGACCACTGAAGGTACC***CCCGTCATACCTACTCCGGCCGCCAACTGGATGGATTG**GCTAACCTGTACGTCATCCGAGTCCCTCAT  GGGAGGGCCCGTCTACAGAACCATCGCATAGACACCTATG*GAGCCT***TTCC**CAGAAGTCTGTGATGTGAAGACATACATGT*CT*G*GGCTGT  TAAAAAGGGACGGAAAAAACGAAAAAAAAAAAAAAAAAAAAAAAAAAATGCTCGCCAGTCATCCCGCTCAACACCCTCCCCGACGGGCTTCCG  CCATCCCCCCCCAAGGGGGGGGAACCAGGGAG | - | - | - |

Legend: (a) Consensus sequences represent the correct order of a sequence. (b) Percentage of identity with other sequences in BLASTn (<https://blast.ncbi.nlm.nih.gov/>).
